# Orphan Nuclear Receptor RORγ Modulates the Genome-Wide Binding of the Cholesterol Metabolic Genes during Mycotoxin-Induced Liver Injury

**DOI:** 10.3390/nu13082539

**Published:** 2021-07-25

**Authors:** Kaiqi Li, Hao Li, Kexin Zhang, Jinying Zhang, Ping Hu, Yanwei Li, Haotian Gu, Hao-Yu Liu, Zhangping Yang, Demin Cai

**Affiliations:** Laboratory of Animal Physiology and Molecular Nutrition, Key Laboratory of Animal Breeding Reproduction and Molecular Design for Jiangsu Province, College of Animal Science and Technology, Yangzhou University, Yangzhou 225009, China; kira_li@foxmail.com (K.L.); 17633532469@163.com (H.L.); kitty20010606@126.com (K.Z.); zhangjinying0317@163.com (J.Z.); huping0514@foxmail.com (P.H.); ywli0805@163.com (Y.L.); guhaotian1998@163.com (H.G.); yzp@yzu.edu.cn (Z.Y.)

**Keywords:** RORγ, nuclear receptor, ChIP-seq, liver injury, mycotoxin, cholesterol biosynthesis

## Abstract

Maintaining lipid homeostasis is crucial to liver function, the key organ that governs the whole-body energy metabolism. In contrast, lipid dysregulation has been implicated in mycotoxin-induced liver injury, by which the pathophysiological regulation and the molecular components involved remain elusive. Here we focused on the potential roles of orphan nuclear receptor (NR) RORγ in lipid programming, and aimed to explore its action on cholesterol regulation in the liver of mycotoxin-exposed piglets. We found that liver tissues were damaged in the mycotoxin-exposed piglets compared to the healthy controls, revealed by histological analysis, elevated seral ALT, AST and ALP levels, and increased caspase 3/7 activities. Consistent with the transcriptomic finding of down-regulated cholesterol metabolism, we demonstrated that both cholesterol contents and cholesterol biosynthesis/transformation gene expressions in the mycotoxin-exposed livers were reduced, including *HMGCS1*, *FDPS*, *SQLE*, *EBP*, *FDFT1* and *VLDLR*. Furthermore, we reported that RORγ binds to the cholesterol metabolic genes in porcine hepatocytes using a genome-wide ChIP-seq analysis, whereas mycotoxin decreased the RORγ binding occupancies genome-wide, especially at the cholesterol metabolic pathway. In addition, we revealed the enrichment of co-factors p300 and SRC, the histone marks H3K27ac and H3K4me2, together with RNA Polymerase II (Pol-II) at the locus of *HMGCS1* in hepatocytes, which were reduced by mycotoxin-exposure. Our results provide a deep insight into the cholesterol metabolism regulation during mycotoxin-induced liver injury, and propose NRs as therapeutic targets for anti-mycotoxin treatments.

## 1. Introduction

Mycotoxins are secondary metabolites produced by mold and fungi (e.g., genera *Aspergillus*, *Penicillium* and *Fusarium* etc.) [1,2,3]. They are naturally present in plants and substrates that are used as foods and feeds, and are therefore almost unavoidable and raise concerns globally [2]. In animal feedstuffs, more than 70% of the samples examined are found to be contaminated with at least one mycotoxin [4]. Therefore, humans and animals are more likely to face co-occurrence of mycotoxins than being challenged with a single one during environmental exposure [5]. Indeed, more than four hundred fungal toxins are being recognized as potential biohazards [6], in which the majority of prevalent mycotoxins are aflatoxins, deoxynivalenol (DON, a type B trichothecene) [3], zearalenone (ZON) and ochratoxins (OTA) [7]. They are known to have a wide range of adverse effects on health, and target multiple organs in mammals [8,9,10], which are probably not mutually exclusive. Aflatoxin B1 (AFB1) is a potent carcinogenic and genotoxic compound that mainly affects the liver, while OTA and ZON induce liver injury and kidney damage in human and animals [7]. DON is shown to cause gastrointestinal (GI) disturbances or immune suppression [9], in addition to compromising liver function [10]. All of this leads to clinical and subclinical symptoms associated with great economic losses [8]. This is particularly true and problematic for the swine industry, given that the pig is a domestic species that is sensitive to the toxic effect of mycotoxins, and is highly susceptible to the contamination [3,4].

Despite the diverse toxic effects that mycotoxin induces in the host, the liver is the key target organ that is responsible for detoxification of ingested xenobiotics. It is reported that mycotoxin OTA induces inflammation in the liver and even hepatocellular carcinoma [11]. Several studies have demonstrated that AFB1 exposure results in liver function damage with significantly increased alkaline phosphatase (ALP) and alanine transaminase (ALT) levels in serum [12,13,14]. These effects may be mediated by skewed lipid metabolism and signaling pathways in hepatocytes [15]. In rats, it is shown that subacute exposure to AFB1 disrupted the hepatic cholesterol metabolism [14]. Ponchon and co-workers suggested that the effects of mycotoxins would impact hepatic cholesterol biosynthesis and subsequently vitamin D activation [16]. Mycotoxin-induced lipid/cholesterol metabolism dysregulation also entails energy imbalance and altered cell membrane integrity [17,18]. For instance, DON treatment changes the lipid profile of cells including decreasing levels of lysolipids, sphingolipids, sterols and free fatty acids (FA), as well as down-regulating bile acid metabolism [17]. However, the molecular network behind this is not fully understood.

Meanwhile, the liver is also the main site of lipid/cholesterol de novo synthesis [15,19], which is commonly known to be controlled by the transcription factor sterol regulatory element-binding protein 2 (SREBP2) [20,21,22]. Often, SREBP2 is cleaved and translocated from endoplasmic reticulum (ER) into the cell nucleus to activate the expression of cholesterol synthesis enzymes of the mevalonate pathway, such as 3-hydroxy-3-methylglutaryl-CoA reductase (HMGCR), squalene monooxygenase (SQLE) and farnesyl pyrophosphate synthase (FDPS) [20,22,23,24,25]. We and others have proved that nuclear receptors (NRs) play cardinal roles in controlling lipid metabolism upon ligand activation [22,26,27], and the NR RORγ may even revoke the SREBP2 regulation of the cholesterol biosynthesis under specific conditions [22,28]. Interestingly, mycotoxin patulin is reported to affect steroidogenesis and the mRNA expression of glucocorticoid receptors (GRs) in vitro in *H295R* cells [29]. It has also been demonstrated to interact with the NR pregnane X receptor (PXR) and the aryl hydrocarbon receptor (AhR) in vitro in human hepatocytes [30]. However, little is known about the relationships between the NR family and mycotoxins in vivo [31]. Given the similarities to humans in metabolism and physiology, the pig is a better-suited in vivo model for human studies compared to other animal models [32,33]. Therefore, this study aimed to investigate the action of NRs in liver pathophysiology in response to mycotoxin contamination, using newborn piglets exposed to a mixture of mycotoxins from environmental and maternal sources. By surveying the local commercial pig farms, we identified feedstuffs contaminated with AFB1, ZON, OTA and DON mycotoxins of lactational sows. Subsequently, we were able to select piglets exposed to these toxins before or at birth, and investigate the roles of NR RORγ in orchestrating the hepatic cholesterol metabolic gene program.

## 2. Materials and Methods

### 2.1. Animals and Sampling

All animal procedures were in accord with and approved by the Animal Ethical Committee of Yangzhou University (202103121). Feeds samples of lactational sows (185 samples/group) from two commercial farms were collected and used to measure the contents of mycotoxins by the competitive enzyme immunoassay using the kits from R-Biopharm AG, including AFB1 (R1211), DON (R5905), ZON (R5505) and OTA (R5402). The sows from the mycotoxin (MTE)-exposed-farm were identified, from which their feeds containing the mean levels of MTE were selected. We further identified newborn piglets from these sows (one per litter of the mean body weight) to investigate their liver function. Serum and liver samples were collected immediately after sacrificing the animals, and were snap-frozen in liquid nitrogen and stored at −80 °C for further analyses.

### 2.2. qRT-PCR Analysis

A total of 2 μg RNA was isolated from the liver tissues. The cDNA was prepared, amplified and measured using SYBR green as previously described. Briefly, the fluorescent values were calculated, and a melting curve analysis was conducted. Fold difference was calculated.

### 2.3. Total Cholesterol and Bile Acid Concentrations Measurement

Total hepatic cholesterol concentration was detected by a commercial total cholesterol assay kit (#E1005; Applygen-Technologies, Inc., Beijing, China). Total bile acid concentrations were measured using enzymatic colorimetric methods with a commercial kit (E003; Nanjing Jian-cheng Bio-engineering Institute, Nanjing, China).

### 2.4. GSEA Analysis of RNA-Seq

The RNA-seq data were downloaded from a previous published dataset [34]. Enrichment Analysis (GSEA v.3.0 version) was performed to rank-genes according to the shrunken limma log_2_ fold changes. The GSEA tools were used in a “pre-ranked” model with default parameters. Gene ontology (GO analysis) was implemented with GO-seq with the Wallenius noncentral hypergeometric distribution in R-package.

### 2.5. Histology Analysis

Sections (5 μm in thickness, 10 sections/sample) from liver samples were fixed in 10% phosphate-buffered formalin and stained using hematoxylin & eosin Y, and visualized by a light microscope using a 20 × objective.

### 2.6. Blood Biochemical Parameters

All biochemical blood parameters used to study the organ functions were performed at the same time to minimize the analytical variabilities, and were detected on a Roche Integra 400 Plus analyzer (Roche Diagnostics).

### 2.7. ChIP-qPCR Measurement and ChIP-Seq Data Analysis

In brief, the liver tissues were minced, crosslinked in 1% formaldehyde for 5 min, and stopped with cold glycine for 5 min. Thereafter, the samples were resuspended in 50 mM HEPES lysis buffer (pH-8.0, 140 mM-NaCl, 1 mM-EDTA, 10%-glycerol, 0.5%-NP-40, 0.25%-Triton X-100). They were then pelleted and washed. Finally, the samples were resuspended in the shearing buffer (pH-8.0, 0.1%-SDS, 1 mM-EDTA, 10 mM-Tris-HCl) and sent for sonication using Covaris E220 following the manufacturer’s instruction.

Next, the crude chromatin fragments were precipitated with the indicated antibodies overnight at 4 °C, and then incubated with protein-G magnetic beads. The purified ChIP-DNA was prepared for ChIP-qPCR and next library generation. Libraries were then quantified using the Agilent bioanalyzer 2100 for sequencing in a single end 50 bp mode on the Illumina HiSeq 2000 Sequencer (BGI, Wuhan). The anti-RORgamma rabbit antibody was used as described previously [35]. Fastq data from ChIP sequence were obtained and were analyzed using the AQUAS pipeline for transcription factors and histone marks.

The RORγ binding site was assigned to the nearest gene. The annotation contains the peaks located in the TSS from −1 kb to +100 bp, TTS from −100 bp to +1 kb, exon (coding region), 5′-UTR and 3′-UTR exon, intronic or intergenic regions. Venn-diagrams of the enriched genomic regions and the associated genes were processed by Intervene. Gene ontology analyses were conducted using Clue-GO and BIN-GO. The motif analysis of DNA fragments was performed by homer script (findMotifGenome.pl) with argument to explore de novo enrichment and the known transcription factor motifs.

### 2.8. Statistical Analysis

All data analyses were processed by GraphPad Prism software 8.0. The data are presented as mean values ± SEM. Statistical analysis was performed using two-tailed Student’s t tests. *p* < 0.05 was considered significant.

## 3. Results

### 3.1. Mycotoxin Exposure Is Associated with Liver Injury in Piglets

**Figure 1:**
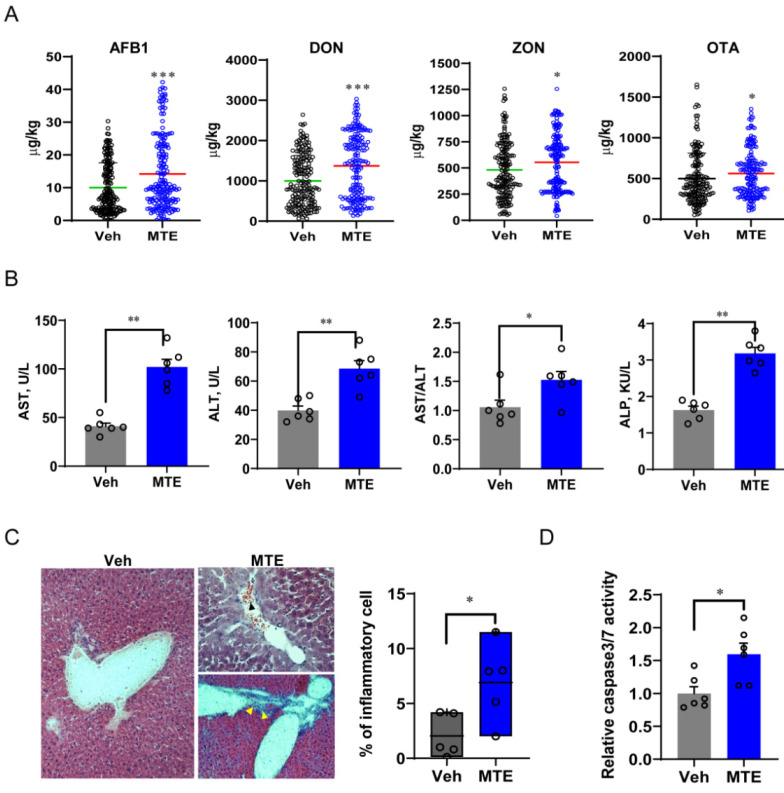
Mycotoxin exposure is associated with liver injury in piglets. (**A**) Feed samples were collected and used to measure the content of mycotoxins by the competitive enzyme immunoassay using the kits from R-Biopharm AG. (**B**) Blood AST (aspartate transaminase), ALT (alanine aminotransferase), and ALP (alkaline phosphatase) concentrations, and the AST/ALT ratio. (**C**) Representative images of liver sections stained with hematoxylin and eosin from the Veh and MTE groups, respectively. The upper right panel shows diffused cell infiltration and blood cell accumulation (black arrow). Yellow arrows (bottom right panel) point to mononuclear cell infiltration at the portal area. (**D**) Analysis of the percentage of inflammatory cells (*n* = 5); hepatocyte apoptosis is reflected by activation of caspase 3/7. The data are shown as the means ± SEM, *n* = 6, * *p* < 0.05, ** *p* < 0.01, *** *p* < 0.001, using ANOVA with Tukey’s post hoc test.

Feed samples of lactational sows (185 samples/group) from two commercial farms were collected and used to evaluate mycotoxin contamination, including the detection of AFB1, DON, ZON and OTA. One farm was found to be contaminated with mycotoxins (MTE-exposed-farm), which exhibited significantly higher levels of all four mycotoxins (Figure 1A). The sows from the MTE-exposed-farm were identified, from which their feeds containing the mean level of MTE were selected. We further selected newborn piglets from these sows (one per litter of the mean body weight) to investigate their liver function. As the most reliable biomarkers of liver injury, blood aspartate transaminase (AST), ALT and ALP levels were measured. Consistent with the higher MTE content in feeds of sow, the serum levels of AST, ALT, and ALP were raised markedly (Figure 1B), suggesting liver damage in the MTE-exposed piglets compared to the healthy controls from the Veh group. This was confirmed by the histopathological examinations, showing infiltrates of mononuclear cells with significantly higher percentage in the MTE-exposed liver tissues (Figure 1C). The inflammatory cells were found both diffusely scattered and surrounding the portal area. In addition, mild liver lesions with disorganization of hepatic cords and blood occlusion were detected (Figure 1C). In parallel, the hepatocyte apoptotic caspase 3/7 activities were obviously increased in the MTE-exposed piglets compared to the Veh group (Figure 1D).

### 3.2. Mycotoxin Exposure Reduces Cholesterol Content and Metabolism

**Figure 2:**
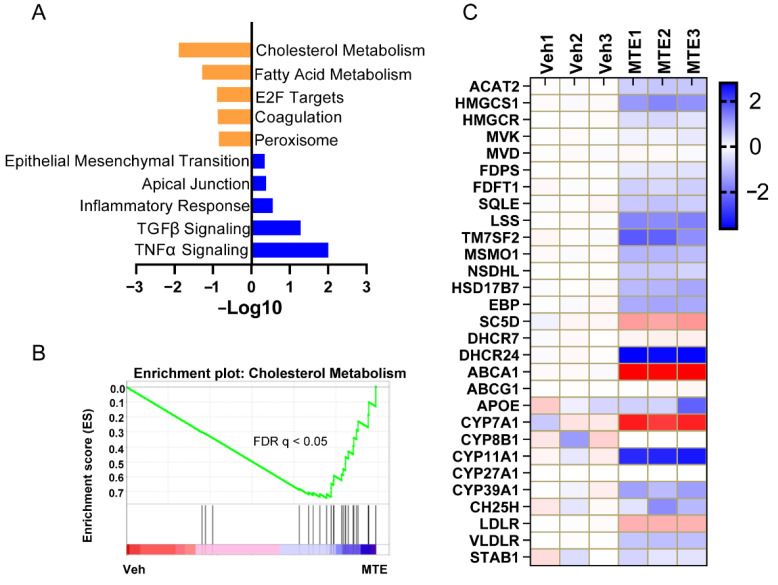
Cholesterol metabolism is susceptible to mycotoxin exposure. (**A**) Differences in activity scores of indicated pathway gene expressions using gene ontology analysis between the Control and the mycotoxin-treated IPEC−J2 cells. (**B**) GSEA plots depicting the enrichment of genes down-regulated in the cholesterol metabolism pathway in porcine IPEC−J2 cells treated by mycotoxin. FDR, false discovery rate. (**C**) Heat−map of the mRNA profile of core cholesterol-metabolism genes.

**Figure 3:**
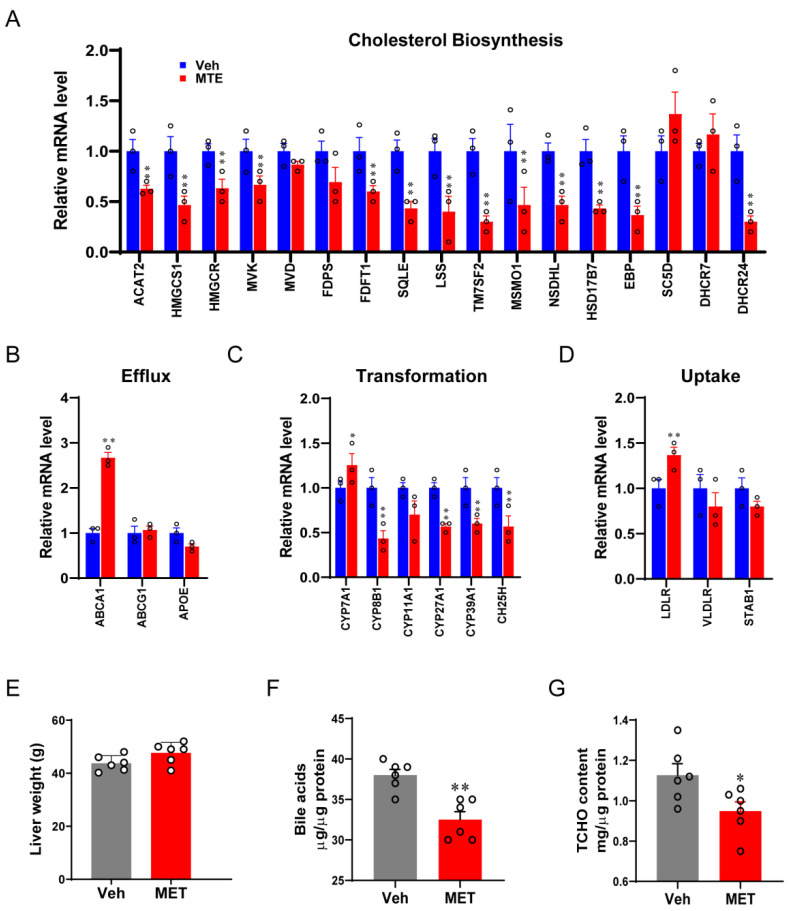
Mycotoxin exposure decreases cholesterol and bile acids synthesis. (**A**–**D**) Cholesterol metabolism gene transcripts measured by qRT-PCR in control or mycotoxin-exposed piglet livers. (**E**) The liver weight was measured in two groups. (**F**) Liver bile acid concentrations and (**G**) total cholesterol (TCHO) contents were tested. The data are shown as the means ± SEM, *n* = 6, * *p* < 0.05, ** *p* < 0.01, using ANOVA with Tukey’s post hoc test.

To explore the key transcriptional program responsible for the mycotoxin contamination in newborn piglets, bioinformatic analysis was first performed using a previously published RNA-seq raw dataset [34] from porcine cells with or without mycotoxin treatment. The gene ontology (GO) analysis of GSEA hallmarks process exhibited that genes enrolled in the cholesterol metabolism were among the top enriched- and downregulated-pathways (Figure 2A,B). Using pathway-focused analysis, it was shown that the majority of genes encoding enzymes that are important for converting acetyl-CoA to cholesterol were reduced in the MTE-exposed cells (Figure 2C). Moreover, we observed that genes involved in the cholesterol-bile acid transformation were also decreased in response to the mycotoxin treatment. In line with the transcriptome results, the cholesterol metabolic gene expressions (Figure 3), in particular cholesterol biosynthesis (Figure 3A) and transformation (Figure 3C), were validated in the livers of MTE-exposed piglets by qRT-PCR analysis, and were shown at lower levels. Furthermore, the cholesterol and bile acid contents were significantly less in the MTE-exposed piglet livers compared to the Veh group (Figure 3F,G). In contrast, the liver weight was not different between the two groups (Figure 3E). Together, these results proved that hepatic cholesterol metabolism of newborn piglets is highly susceptible to mycotoxin exposure.

### 3.3. Mycotoxin Exposure Reduces the RORγ Genome-Wide Binding on Genes of Cholesterol Metabolic Pathways

**Figure 4:**
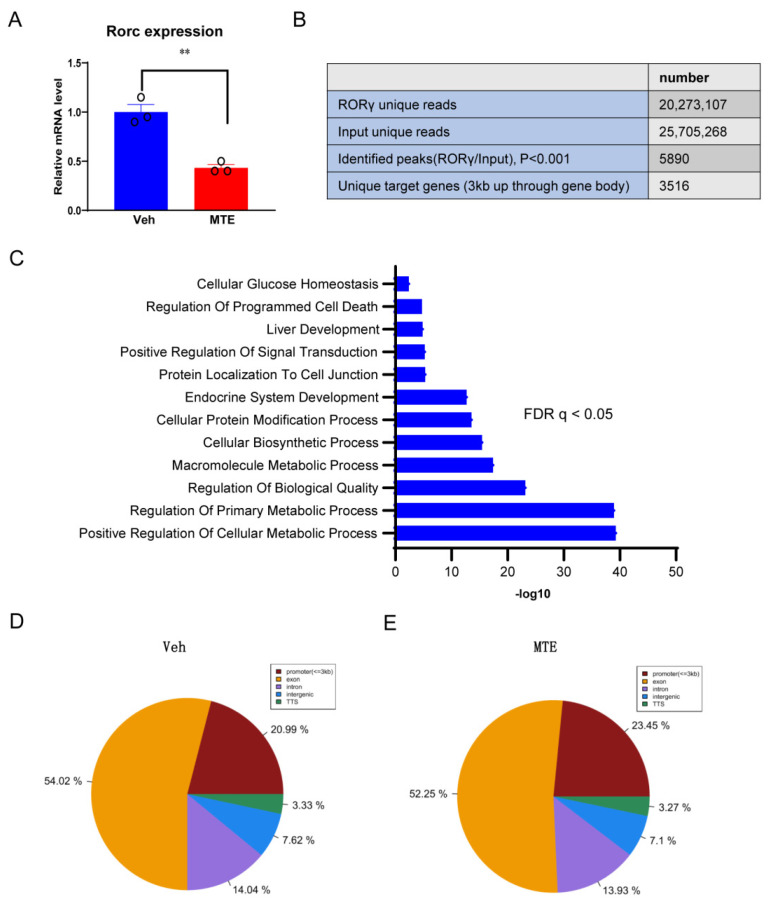
Genome-wide ChIP-seq analysis of RORγ enrichments in piglet liver. (**A**) RORγ gene mRNA analyzed by qRT-PCR in control or mycotoxin-exposed piglet liver. (**B**) Summary of RORγ ChIP-Seq analysis and porcine liver chromatin. (**C**) GO analysis of RORγ ChIP-seq peak-linked genes. (**D**,**E**) The binding sites were localized within intergenic regions, introns, exon, transcriptional start site (TSS), and within a 3 kb region of the promoter in the livers.

Nuclear receptors exert crucial functions in the modulation of cholesterol metabolism. Importantly, we have demonstrated previously that the orphan RORγ acts as a novel transcription factor of cholesterol de novo synthesis in porcine liver organoids [28]. Herein, we first studied the gene expression of RORγ, and revealed that it was downregulated in the MTE-exposed piglet liver compared to the Veh group (Figure 4A). To delineate the underlying mechanisms, we performed RORγ ChIP-seq analysis in the livers of piglets. This analysis identified 5890 RORγ binding sites (Figure 4B), and the displayed ChIP-seq peaks showed that cellular metabolic processes were among the significantly enriched programs using a gene ontology analysis (Figure 4C). These data also demonstrated that the binding sites were localized within intergenic regions (7.62%), introns (14.04%), exon (54.02%), transcriptional start site (TSS, 3.33%), and within a 3 kb region of the promoter (20.99%) in the livers of healthy piglets (Figure 4D). In contrast, when exposed to MTE, the locus was switched to intergenic regions (7.10%), introns (13.93%), exon (52.25%), TSS (3.27%), and promoter (23.45%) (Figure 4E). The switched RORγ genome-wide binding, especially in the exon and the promoter regions implied that the functions of RORγ may be altered due to MTE-exposure.

**Figure 5:**
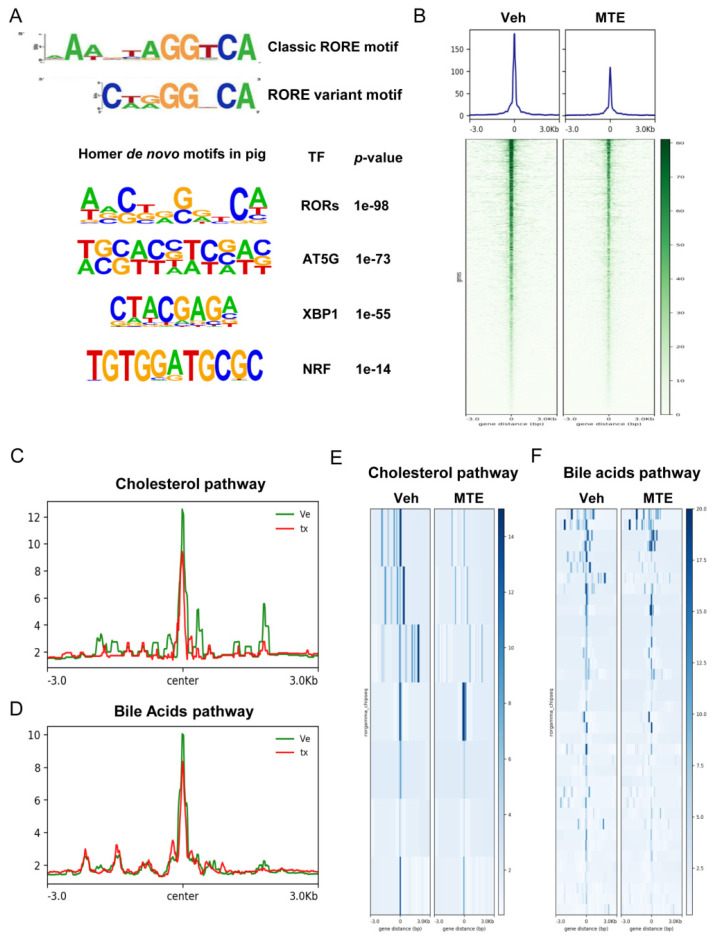
Mycotoxin exposure reduces RORγ binding on cholesterol and bile acids pathways. (**A**) Homer and known transcription factor (TF) motifs analysis for RORγ enriched peaks. (**B**) Genome−wide ChIP−Seq profiles and heat−map of RORγ signal intensity within +/−3 kb regions around the peak center. (**C**,**D**) ChIP−Seq profiles of RORγ binding on genes enrolled in cholesterol−bile acids metabolism pathway. (**E**,**F**) Heat-map of ChIP−Seq signal intensity as (**C**,**D**).

Moreover, the deep TF motif analysis of RORγ-peaked regions identified ROR response elements (ROREs) as the top-ranking motif. We also identified porcine RORE motif *GGGTCA* as a variant RORE motif using the MEME program (Figure 5A). Mycotoxin-exposure drastically reduced the genome-wide RORγ association to its targets (Figure 5B). In line with the critical function of RORγ in modulating cholesterol-bile acids metabolic program, MTE-exposure markedly reduced the RORγ binding to cholesterol (Figure 5C,E) and bile acids (Figure 5D,F) program gene loci. Together, the data indicated that loss of RORγ occupancy is a pivotal event for MTE-triggered dysfunctional cholesterol metabolism.

### 3.4. Loss of RORγ Binding at the Enhancers and the Promoters of Cholesterol Metabolic Genes

**Figure 6:**
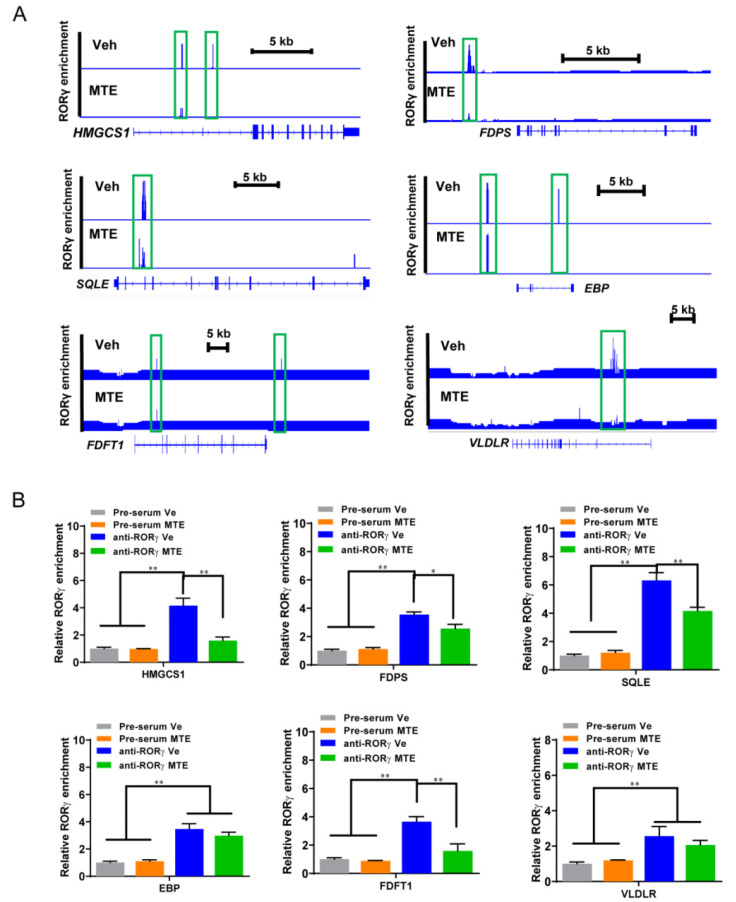
Loss of RORγ binding at the enhancers and promoters of cholesterol metabolic genes. (**A**) Signal visualization of RORγ ChIP-seq at indicated cholesterol metabolism genes. (**B**) ChIP-qPCR analysis of hepatic RORγ binding in control or mycotoxin piglets. The data are shown as the means ± SD, *n* = 3. The experiments were repeated three times. * *p* < 0.05, ** *p* < 0.01.

Having shown the crucial action of RORγ in the regulation of the cholesterol metabolic pathway, we next examined which genes were vulnerable to MTE-exposure. Concomitant with the down-regulated transcripts involved in cholesterol metabolism, the results showed a dramatic loss of RORγ binding at the major gene enhancers of including *HMGCS1*, *FDPS*, *SQLE*, *EBP*, *FDFT1* and *VLDLR* in ChIP-seq analyses (Figure 6A). To verify and quantify these results, a ChIP-qPCR analysis was performed. As shown in Figure 6B, the cholesterol biosynthesis genes all displayed lower RORγ enrichments on the specific binding sites, except for *EBP.* The results confirmed that MTE-exposure causes cholesterol content reduction by targeting its transcription inactivation.

### 3.5. Mycotoxin Exposure Modifies RORγ-Mediated Histone Modifications

**Figure 7:**
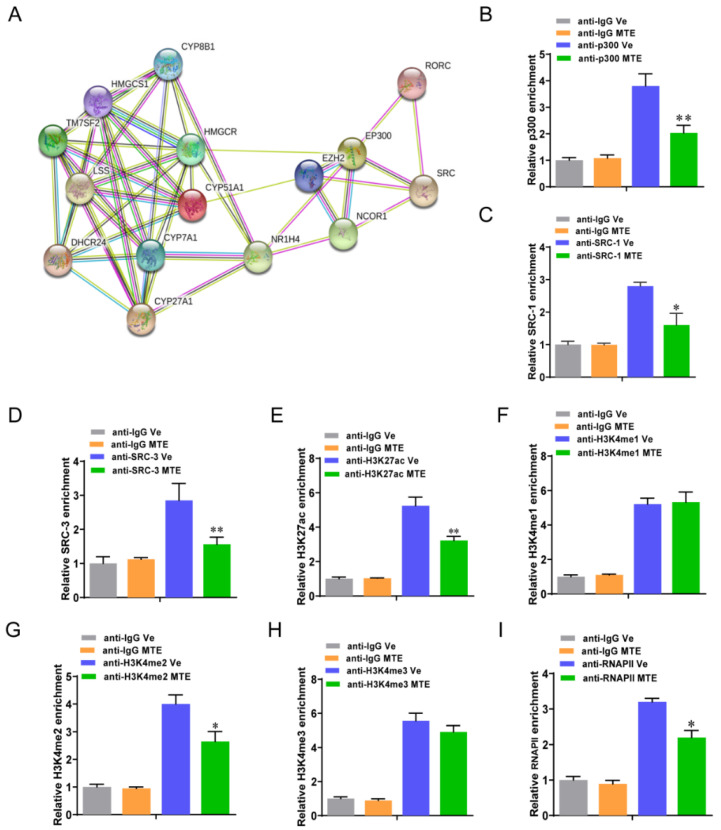
Mycotoxin exposure modulated RORγ-associated histones modification. (**A**) STRING analysis of candidate co-factors of RORγ transcriptional regulation. (**B**–**D**) ChIP-qPCR analysis of the relative enrichments of p300, SRC-1 and SRC-3 at the locus of *HMGCS1*. (**E**–**I**) ChIP-qPCR analysis of the indicated enrichments of H3K27ac, H3K4me1/2/3 and RNA Polymerase II. Data were presented as means ± SD, * *p* < 0.05, ** *p* < 0.01.

Finally, we studied whether co-factors or histone marks assisted the roles of RORγ in the programming of cholesterol metabolism of MTE-exposed piglets. The putative co-factors p300, SRC, and NCOR1 were predicted by STRING-ELIXIR database (Figure 7A). As we observed that the occupancies of p300, SRC1 and SRC3 were significantly reduced at the *HMGCS1* enhancer in the MTE-exposed livers (Figure 7B–D). We next conducted a ChIP-qPCR analysis to measure the histone-active marks H3K27ac and H3K4me1/2/3 at the *HMGCS1* locus. We found that MTE-exposure significantly reduced the H3K27ac (Figure 7E) and H3K4me2 (Figure 7G) enrichments, but not H3K4me1/3 (Figure 7F,H). In association with the loss of mRNA expression of cholesterol metabolism genes, the promoter enrichments of RNA Polymerase II (Pol-II) were found to be diminished accordingly in the MTE-exposed group (Figure 7I). Collectively, these data indicated that RORγ-linked chromatin remodeling of cholesterol metabolism genes was responsible for MTE-exposure in the livers of newborn piglets.

## 4. Discussion

Mycotoxins are nearly all toxic and are readily taken up by animals and humans, with the highest absorption rate of 66% in pigs [36]. Lipid dysregulation has been implicated in mycotoxin-induced liver injury. Since the NR RORγ is involved in the maintenance of cholesterol homeostasis, its interaction with mycotoxin in the livers of pigs is of great interest. Herein, we surveyed the local commercial farms, identified feed samples from lactational sows that were contaminated with a mixture of four mycotoxins, i.e., AFB1, DON, ZON and OTA. By selecting the corresponding offspring, we were able to take a deep insight into the liver function of newborn piglets subjected to environmental and maternal sources of mycotoxin challenges. Indeed, we found liver injury of piglets at birth due to mycotoxin exposure. We uncovered the altered hepatic cholesterol biosynthesis program, including reduced expression of key genes *HMGCS1*, *FDPS*, *SQLE*, *EBP*, *FDFT1* and *VLDLR*, in parallel with changes in bile acid transformation gene expressions. More importantly, we revealed that the orphan NR RORγ is the driver of this lipid metabolic reprogramming in the livers of piglets in vivo using a genome-wide ChIP-seq analysis for the first time. During which, the associated local histone modifications of RORγ binding on the target locus of *HMGCS1* was demonstrated.

The liver represents a crucial detoxifier of the host, constantly filtering environmental substances. Therefore, major hepatotoxic effects have been detected in domestic animals challenged by mycotoxins [37]. Like our in vivo study, mycotoxin-exposure resulted in increased serum levels of AST, ALT and ALP in piglets. These enzymes are localized in the cytoplasm of hepatocytes, and are released into the blood stream during liver injury [5]. Similarly, several studies have shown that experimental treatment of AFB1 and DON mixture reduces the growth performance of pigs and causes liver damage [38,39]. Metabolites derived from aflatoxins in the liver have been reported to also disrupt blood coagulation [40], which is consistent with the observed blood occlusion in liver histology of our MTE-exposed piglets and the reduced coagulation gene expression. When the ingested mycotoxin is at higher levels, hepatocyte apoptosis and lobular necrosis may occur [40], and further affect the bile duct in conjunction, as shown by the reduced bile acid content and synthesis gene expression found in our study in mycotoxin-exposed piglets. A positive correlation between AFB1-increased ALP and bilirubin has been reported, suggesting a hepatobiliary action induced by mycotoxins [41]. In addition, we also found an increased caspase 3/7 activity in the liver, in parallel with the increased gene expression of TGFβ signaling, which may be a reflection of the prominent apoptosis and possible fibrosis in mycotoxin-injured livers [42].

The reported liver damage induced by mycotoxin-exposure in piglets is linked with decreased concentrations of cholesterol in the current study, as it is mainly synthesized by hepatocytes [43]. Accordingly, Holanda and co-workers showed that a mycotoxin-contaminated diet reduces blood cholesterol levels in newly weaned piglets [3]. In contrast, food contaminated with DON causes non-alcoholic fatty liver disease (NAFLD) in mice due to metabolic impairments. Notably in this study, the DON associated lipogenesis (*FASN* and *SCF1*) and cholesterogenesis (*HMGCR*) pathways were first upregulated, but then followed by a down-regulation [44], implying a possible exhaustion and fat degeneration caused by mycotoxins. We have demonstrated that mycotoxin intoxication fundamentally alters the hepatic cholesterol biosynthesis gene program. More importantly, we revealed that it is driven by RORγ, where the expression is decreased in the presence of mycotoxins. This NR expressed in hepatocytes exerts a critical role in maintaining cholesterol homeostasis. The reduced total cholesterol contents were due to activation of the key enzymes, including primarily HMGCS, as well as FDPS, SQLE and FDFT1, which are involved in converting acetyl-CoA to cholesterol. It has been recognized that SREBP2 is the classic controller for cholesterol de novo synthesis gene activation [45,46,47], including *HMGCS1*. Our previous studies revealed that the NR RORγ exhibits a predominant action over that of SREBP2 in programming cholesterol biosynthesis in tumor cells [22] and in porcine liver organoids [28], respectively, which is in agreement with our present findings. By further validating the downstream factors, we revealed that RORγ binds to the cholesterol metabolic genes in porcine hepatocytes using a genome-wide ChIP-seq analysis, in which RORγ binding occurs at the enhancers and the promoters of target loci. We showed clearly that mycotoxin-exposure decreases RORγ binding on *HMGCS1*, including the decreased enrichments of p300 and SRC together with the histone marks H3K27ac and H3K4me2, as well as RNA Pol-II. However, the RORγ-driven epigenetic regulation is not necessarily the only mechanism that explains the altered hepatic expression of cholesterol metabolic genes. The involvement of other nuclear receptors and their crosstalk cannot be ruled out. Furthermore, we postulate that mycotoxin-exposure-induced liver injury in neonates may be correlated with one’s health or susceptibility to disease in later-life, which warrants further longitudinal investigations.

## 5. Conclusions

In conclusion, our study has identified a novel mechanism linking mycotoxin-exposure-induced liver injury to the orphan NR RORγ-driven genome-wide binding of cholesterol metabolism in newborn piglets. Mycotoxin-exposure, even indirectly from environmental and maternal sources, alters the hepatic cholesterol metabolic gene program, bile acid synthesis, and the transcription activity of the master regulator RORγ, leading to a decrease in total cholesterol contents and an increase in hepatocyte apoptosis, and ultimately induces liver damage. The findings in piglets may help to understand the role of NRs in neonatal programming of cholesterol metabolism in humans, given their similarities in physiology and metabolism. Our results shed light on the pathophysiological regulation of livers in response to mycotoxins and NRs as potential therapeutic targets, and open a new avenue for prevention or intervention of mycotoxicosis.

## Data Availability

The data that support the findings of this study are available on request from the corresponding author.
